# Associations Between Unprocessed Red Meat and Processed Meat With Risk of Recurrence and Mortality in Patients With Stage III Colon Cancer

**DOI:** 10.1001/jamanetworkopen.2022.0145

**Published:** 2022-02-22

**Authors:** Erin L. Van Blarigan, Fang-Shu Ou, Tiffany M. Bainter, Charles S. Fuchs, Donna Niedzwiecki, Sui Zhang, Leonard B. Saltz, Robert J. Mayer, Alexander Hantel, Al B. Benson, Daniel Atienza, Michael Messino, Hedy L. Kindler, Alan P. Venook, Shuji Ogino, Hanna K. Sanoff, Edward L. Giovannucci, Kimmie Ng, Jeffrey A. Meyerhardt

**Affiliations:** 1 Department of Epidemiology and Biostatistics, University of California San Francisco, San Francisco; 2 Department of Urology, University of California at San Francisco, San Francisco; 3 Alliance Statistics and Data Management Center, Mayo Clinic, Rochester, Minnesota; 4 Yale Cancer Center, Yale School of Medicine, New Haven, Connecticut; 5 Department of Biostatistics and Bioinformatics, Duke University School of Medicine, Durham, North Carolina; 6 Johns Hopkins Bloomberg School of Public Health, Baltimore, Maryland; 7 Memorial Sloan Kettering Cancer Center, New York, New York; 8 Dana-Farber Cancer Institute, Boston, Massachusetts; 9 Edward Hematology Oncology Group, Naperville, Illinois; 10 Robert H. Lurie Comprehensive Cancer Center, Northwestern University, Chicago, Illinois; 11 Virginia Oncology Associates, Norfolk; 12 Southeast Clinical Oncology Research Consortium, Mission Hospitals, Inc, Asheville, North Carolina; 13 University of Chicago Comprehensive Cancer Center, Chicago, Illinois; 14 Helen Diller Family Comprehensive Cancer Center, San Francisco, California; 15 Division of Hematology/Oncology, Department of Medicine, University of California at San Francisco, San Francisco; 16 Program in Molecular Pathology Epidemiology, Department of Pathology, Brigham and Women’s Hospital and Harvard Medical School, Boston, Massachusetts; 17 Department of Epidemiology, Harvard T.H. Chan School of Public Health, Boston, Massachusetts; 18 Broad Institute of Massachusetts Institute of Technology and Harvard, Cambridge; 19 University of North Carolina Lineberger Comprehensive Cancer Center, Chapel Hill; 20 Channing Division of Network Medicine, Department of Medicine, Brigham and Women’s Hospital and Harvard Medical School, Boston, Massachusetts; 21 Department of Nutrition, Harvard T.H. Chan School of Public Health, Boston, Massachusetts

## Abstract

**Importance:**

The American Cancer Society and American Institute for Cancer Research recommend that cancer survivors limit intake of red and processed meats. This recommendation is based on consistent associations between red and processed meat intake and cancer risk, particularly risk of colorectal cancer, but fewer data are available on red and processed meat intake after cancer diagnosis.

**Objectives:**

To examine whether intake of unprocessed red meat or processed meat is associated with risk of cancer recurrence or mortality in patients with colon cancer.

**Design, Setting, and Participants:**

This prospective cohort study used data from participants with stage III colon cancer enrolled in the Cancer and Leukemia Group B (CALGB 89803/Alliance) trial between 1999 and 2001. The clinical database for this analysis was frozen on November 9, 2009; the current data analyses were finalized in December 2021.

**Exposures:**

Quartiles of unprocessed red meat and processed meat intake assessed using a validated food frequency questionnaire during and 6 months after chemotherapy.

**Main Outcomes and Measures:**

Hazard ratios (HRs) and 95% CIs for risk of cancer recurrence or death and all-cause mortality.

**Results:**

This study was conducted among 1011 patients with stage III colon cancer. The median (IQR) age at enrollment was 60 (51-69) years, 442 patients (44%) were women, and 899 patients (89%) were White. Over a median (IQR) follow-up period of 6.6 (1.9-7.5) years, we observed 305 deaths and 81 recurrences without death during follow-up (386 events combined). Intake of unprocessed red meat or processed meat after colon cancer diagnosis was not associated with risk of recurrence or mortality. The multivariable HRs comparing the highest vs lowest quartiles for cancer recurrence or death were 0.84 (95% CI, 0.58-1.23) for unprocessed red meat and 1.05 (95% CI, 0.75-1.47) for processed meat. For all-cause mortality, the corresponding HRs were 0.71 (95% CI, 0.47-1.07) for unprocessed red meat and 1.04 (95% CI, 0.72-1.51) for processed meat.

**Conclusions and Relevance:**

In this cohort study, postdiagnosis intake of unprocessed red meat or processed meat was not associated with risk of recurrence or death among patients with stage III colon cancer.

## Key Points

**Question:**

Among patients with colon cancer, is consumption of unprocessed red meat or processed meats after diagnosis associated with higher risk of recurrence and mortality?

**Findings:**

In this cohort study of 1011 patients with colon cancer, intake of unprocessed red meat or processed meat was not associated with risk of cancer recurrence or death (disease-free survival) or overall mortality.

**Meaning:**

These findings suggest that unprocessed red meat and processed meat intakes after colon cancer diagnosis are not associated with time to recurrence or death.

## Teaser

This cohort study examines whether unprocessed red meat or processed meat intake is associated with risk of cancer recurrence or mortality in patients with stage III colon cancer.

## Introduction

The American Cancer Society (ACS) and American Institute for Cancer Research/World Cancer Research Fund (AICR/WCRF) recommend that cancer survivors limit intake of red and processed meats.^1,2^ This recommendation is based on associations between red and processed meat intake and cancer risk, particularly risk of colorectal cancer. Less is known regarding whether red and processed meat intake after cancer diagnosis is associated with recurrence or mortality.

In 2019, Carr et al^3^ reported that red and processed meat intake before diagnosis was not associated with colorectal cancer survival in a pooled analysis of 10 studies, including 7627 patients with colorectal cancer. For postdiagnosis intake, 2 studies analyzed in a literature review^4^ reported no association between postdiagnostic red and processed meat intake and cancer-specific or all-cause mortality. In addition, we previously reported^5^ that total combined red and processed meat intake was not associated with shorter survival in secondary analyses when examining the ACS guideline score in relation to disease-free and overall survival among patients with stage III colon cancer in the Cancer and Leukemia Group B (CALGB) 89803 cohort.

Nevertheless, avoidance of red and processed meat remains one of the few dietary recommendations given to patients with colorectal cancer. Further data are needed to understand whether this recommendation is warranted. Thus, we prospectively examined unprocessed red meat and processed meat intake after diagnosis in association with risk of cancer recurrence or death and all-cause mortality among patients with stage III colon cancer. We hypothesized that unprocessed red meat intake would not be associated with risk of colon cancer recurrence or mortality. This analysis adds to previously reported data from this cohort by examining unprocessed red meat and processed meats separately and adjusting for additional dietary factors.

## Methods

### Study Population

This analysis was conducted among 1095 individuals with stage III colon cancer who consented and enrolled in the National Cancer Institute–sponsored CALGB 89803 adjuvant chemotherapy trial between 1999 and 2001 and completed a survey midway through treatment (approximately 3 months after diagnosis).^6^ A second survey was administered 6 months after treatment (approximately 15 months after diagnosis); 981 individuals completed the second survey. This study was conducted in accordance with federal and institutional ethical guidelines and was approved by each site’s institutional review board. Each participant signed an institutional review board–approved, protocol-specific informed consent document. Details of the trial and participants who completed the surveys have been reported elsewhere.^5^ CALGB is now part of Alliance for Clinical Trials in Oncology. This cohort study follows the Strengthening the Reporting of Observational Studies in Epidemiology (STROBE) reporting guideline.

To be included in this analysis, participants had to complete the first survey prior to cancer recurrence (1087 participants). To reduce measurement error in estimates of diet, we excluded 46 individuals who had an estimated intake less than 500 or greater than 3500 kcal per day for women or less than 800 or greater than 4200 kcal per day for men or were missing more than 70 items on the diet survey. We also excluded 30 participants who had recurrent disease or died within 3 months after completing the first survey to limit potential reverse causation.

### Dietary Assessment

The surveys included a validated food frequency questionnaire (FFQ) at both points.^7^ The FFQ queried usual intake over the past 3 months of more than 130 items. Participants were asked how often, on average, they consumed a specified portion of the food items. Unprocessed red meat included hamburger, lean or extra lean (1 patty); hamburger, regular (1 patty); beef, pork, or lamb as a sandwich or mixed dish (eg, stew, casserole, or lasagna); pork as a main dish (eg, ham or chops, 4-6 oz [113-170 g]); and beef or lamb as a main dish (eg, steak or roast; 4-6 oz [113-170 g]). Processed meats included beef or pork hot dogs (1 piece); chicken or turkey hot dogs (1 piece); salami, bologna, or other processed meat sandwiches; processed meats (eg, sausage or kielbasa; 2 oz [57 g] or 2 small links); and bacon (2 slices). After excluding individuals with extreme energy intakes and/or missing more than 70 items on the FFQ, we assumed any remaining missing items were not eaten. We calculated cumulative mean postdiagnostic intakes from the 2 FFQs and weighted exposures proportional to follow-up time, as described elsewhere.^6^ If an individual did not complete the second FFQ, the first FFQ was used to assign exposure.

### Covariate Ascertainment

Participants were asked to self-report their sex as male or female. Race and ethnicity were assessed in this study as mandated by the National Institutes of Health. Race and ethnicity were abstracted from the medical record by study staff at enrollment. Three groups were used for this analysis: White race, Black race, and other race or ethnicity, which included Hispanic, Asian, Native Hawaiian, Native American, Indian, other, or multiple. Three individuals with unknown race were categorized in the other race or ethnicity group. Other health behaviors assessed on the survey included current weight and height, smoking history, aspirin use, physical activity, and vitamins and mineral supplements. We calculated body mass index (BMI; weight in kilograms divided by height in meters squared) from height and weight. A validated physical activity survey asked participants to report their average time per week spent at each of 9 recreational activities in the past 2 months. Each activity was assigned a metabolic equivalent task (MET) value,^8^ which we multiplied by the time spent engaged in that activity and summed across all activities to estimate total MET-hours per week of physical activity. If a participant skipped a recreational activity on the survey, they were assumed not to engage in that activity.

There were limited missing data on covariates, as shown in Table 1. We used missing indicators to adjust for missing categorical covariates. One woman was missing BMI at enrollment; this individual was assigned the sex-specific mean BMI of 27.7. Six individuals were missing physical activity at enrollment; these individuals were assigned the sex-specific mean of 9.2 total MET-hours per week for women and 15.7 total MET-hours per week for men. Three individuals who had returned both the first and second survey did not complete the physical activity questions on the second survey; we carried forward the nonmissing baseline physical activity data for these 3 individuals. Finally, 1 person who completed both the first and second survey did not complete the weight question on the second survey; we carried forward their nonmissing baseline BMI.

**Table 1.** Characteristics of 1011 Patients With Stage III Colon Cancer, by Postdiagnostic Intake of Unprocessed Red Meat and Processed Meat

Characteristic	Patients, No. (%)			
	Unprocessed red meat		Processed meats	
	Quartile 1 (n = 252)	Quartile 4 (n = 253)	Quartile 1 (n = 251)	Quartile 4 (n = 253)
Meat intake, median (range), No. of servings/wk	1.5 (0-2.0)	6.9 (5.3-15.1)	0.7 (0-1.1)	5.2 (3.8-30.0)
Age, median (IQR), y	62 (53-70)	60 (51-67)	60 (53-68)	60 (51-69)
Sex				
Male	118 (47)	169 (67)	96 (38)	191 (75)
Female	134 (53)	84 (33)	155 (62)	62 (25)
Race				
Black	27 (11)	11 (4)	8 (3)	32 (13)
White	206 (82)	234 (92)	225 (90)	211 (83)
Other^a^	19 (8)	8 (3)	18 (7)	10 (4)
Performance status				
Fully active	183 (73)	192 (76)	193 (77)	180 (71)
Restricted in strenuous activity	60 (24)	56 (22)	53 (21)	68 (27)
Missing	9 (4)	5 (2)	5 (2)	5 (2)
Bowel wall invasion				
T1-T2	38 (15)	33 (13)	41 (16)	27 (11)
T3-T4	192 (76)	204 (81)	190 (76)	212 (84)
Missing	22 (9)	16 (6)	20 (8)	14 (6)
Positive lymph nodes				
1-3 (N1)	152 (60)	149 (59)	161 (64)	168 (66)
≥4 (N2)	91 (36)	98 (39)	85 (34)	81 (32)
Missing	9 (4)	6 (2)	5 (2)	4 (2)
Clinical bowel abnormality				
Perforation	12 (5)	12 (5)	12 (5)	11 (4)
Obstruction	54 (21)	55 (22)	53 (21)	63 (25)
Grade of differentiation				
Well	14 (6)	17 (7)	16 (6)	17 (7)
Moderate	173 (69)	162 (64)	171 (68)	180 (71)
Poor	55 (22)	69 (27)	58 (23)	52 (21)
Missing	10 (4)	5 (2)	6 (2)	4 (2)
Treatment group				
Fluorouracil plus leucovorin	133 (53)	133 (53)	140 (56)	130 (51)
Irinotecan, fluorouracil, and leucovorin	119 (47)	120 (47)	111 (44)	123 (49)
Smoking status at enrollment				
Current	19 (8)	35 (14)	14 (6)	38 (15)
Past	110 (44)	117 (46)	100 (40)	104 (41)
Never	120 (48)	99 (39)	133 (53)	108 (43)
Missing	3 (1)	2 (1)	4 (2)	3 (1)
Aspirin user	20 (8)	24 (9)	19 (8)	25 (10)
Body mass index at enrollment, median (IQR)^b^	26.6 (23.5-30.6)	27.6 (24.6-32.3)	25.8 (23.0-30.6)	27.5 (24.2-31.4)
Total physical activity at enrollment, median (IQR), metabolic equivalent task h/wk	4.0 (0.6-13.6)	6.0 (1.9-18.0)	4.2 (1.0-10.7)	6.1 (0.9-17.2)
Nutritional intake, median (IQR)				
Carbohydrate, g/d	275 (247-303)	233 (207-254)	270 (241-294)	240 (215-262)
Protein, g/d	78 (68-90)	88 (80-98)	86 (74-97)	81 (73-89)
Total fat, g/d	65 (57-76)	76 (70-83)	65 (56-74)	77 (70-85)
Alcohol, g/d	0.3 (0-3.5)	1.1 (0-7.7)	0.9 (0-4.5)	0.7 (0-5.5)
Vitamin D, IU/d	425 (187-642)	274 (172-482)	433 (207-642)	279 (150-474)
Dark fish, No. of servings/wk	0.2 (0-0.5)	0.2 (0-0.5)	0.2 (0-0.5)	0.2 (0-0.4)
Cereal fiber, g/d	6.2 (4.4-8.1)	5.0 (4.0-6.3)	6.6 (4.6-8.0)	4.8 (3.9-6.0)

^a^ Other race included Hispanic, Asian, Native Hawaiian, Native American, Indian, other, or multiple race. Three people were missing race information and were included in the other race category.

^b^ Body mass index is calculated as weight in kilograms divided by height in meters squared.

### Outcome Ascertainment

Our primary end point was the combined end point of documented disease recurrence, occurrence of a new primary colon tumor, or death from any cause.^6^ Secondarily, we examined risk of overall mortality. These 2 end points were the primary end points of the parent trial.^9^ As part of the parent trial, participants were followed up for 7 years after end of treatment for events of recurrence and mortality; events of first recurrence were confirmed by biopsy when possible. The Alliance Statistics and Data Center collected the data; the clinical database was frozen on November 9, 2009.

### Statistical Analysis

We used Cox proportional hazards regression to estimate hazard ratios (HRs) and 95% CIs for associations between unprocessed red meat and processed meat intake and risk of colon cancer recurrence and mortality.^10,11,12^ Time-to-event variables were defined from completion of the first FFQ to cancer recurrence or death, whichever came first, when examining the combined end point of disease-free survival or death when examining all-cause mortality. We analyzed unprocessed red meat and processed meat intake in quartiles to avoid assumptions of linearity and to reduce the impact of extreme values. Our basic model was adjusted for age, sex, race, and energy intake. The energy intake covariate was allowed to vary over time in the model, so energy intake was updated at the second FFQ if available. Our second model also included treatment group, number of positive lymph nodes, depth of invasion through the bowel wall, tumor grade, perforation, obstruction, baseline performance status, BMI, leisure-time physical activity, smoking, aspirin use and intake of alcohol, vitamin D, carbohydrates, dark fish, cereal fiber, high-fat dairy, and eggs. The BMI and physical activity variables were updated at the second FFQ, if available (time-varying). We considered adjustment for sugar-sweetened and artificially sweetened beverages, nuts, coffee, poultry, and low-fat dairy, but these did not meaningfully change the exposures’ HRs and were omitted from final models.

To explore effect modification, we created cross-product terms between the exposures and potential modifiers (age <70 vs ≥70 years old^3^; male vs female sex; BMI <30 vs ≥30), included the cross-products in our multivariable models, and examined Wald test *P* values. The effect modification analysis was considered exploratory and not adjusted for multiple testing. We also conducted 2 sensitivity analyses: first, we excluded 44 patients who had an event within 6 months of the first FFQ to further assess reverse causation, and second, we used the second survey only to assess exposure and accrued follow-up from the second survey forward to reduce measurement error in usual postdiagnosis diet on the first survey from potential changes in diet due to treatment. This analysis included 805 participants who had not experienced disease recurrence before completing the second questionnaire, of whom 227 experienced recurrence or death (169 deaths) over a median (IQR) follow-up of 6.2 (2.9-6.7) years after the second questionnaire. Analyses were conducted using SAS statistical software version 9.4 (SAS Institute), and 2-sided *P* < .05 was considered significant. The current data analyses were finalized in December 2021.

## Results

This study was conducted among 1011 patients with stage III colon cancer. The median (IQR) age at enrollment was 60 (51-69) years; 442 participants (44%) were women, and 899 (89%) were White. We observed 305 total deaths and 81 events of recurrence without death (386 events combined) over a median (IQR) follow-up period of 6.6 (1.9-7.5) years.

Characteristics of the cohort are shown in Table 1. Patients in the fourth quartile of unprocessed red meat were more likely than those in the first quartile to be male (169 patients [67%] vs 118 patients [47%]); to be White (234 patients [92%] vs 206 patients [82%]); to have been diagnosed with stage T3 or T4 cancer (204 patients [81%] vs 192 patients [76%]); to have poorly or undifferentiated disease (69 patients [27%] vs 55 patients [22%]); to have higher median (IQR) BMI (27.6 [24.6-32.3] vs 26.6 [23.5-30.6]), higher physical activity (6.0 vs 4.0 MET-hours per week), and higher intake of protein (88 vs 78 g/d), fat (76 vs 65 g/d), and alcohol (1.1 vs 0.3 g/d); and to have lower intake of carbohydrates (233 vs 275 g/d), vitamin D (274 vs 425 IU/d), and cereal fiber (5.0 vs 6.2 g/d) (Table 1). People in the fourth quartile of processed meat intake were more likely than those in the first quartile to be male (191 patients [75%] vs 96 patients [38%]); to be Black (32 patients [13%] vs 8 patients [3%]); to be current smokers (38 patients [15%] vs 14 patients [6%]); to have higher median (IQR) BMI (27.5 [24.2-31.4] vs 25.8 [23.0-30.6]); to be restricted in strenuous activity at enrollment (68 patients [27%] vs 53 patients [21%]); to have been diagnosed with stage T3 or T4 disease (212 patients [84%] vs 190 patients [76%]); to have reported lower median intake of carbohydrates (240 vs 270 g/d), protein (81 vs 86 g/d), vitamin D (279 vs 433 IU/d), and cereal fiber (4.8 vs 6.6 g/d); and to have reported higher intake of fat (77 vs 65 g/d). Of note, unprocessed red meat and processed meat were moderately correlated with each other (*r* = 0.41;* P* < .001) and with carbohydrate intake (*r* = 0.41 for unprocessed red meat and *r* = −0.29 for processed meat;* P* < .001).

In our first model, higher unprocessed red meat intake was associated with lower risk of colon cancer recurrence and death (Table 2). Participants in the fourth quartile of unprocessed red meat intake had a 42% lower risk of cancer recurrence or death compared with participants in the lowest quartile (HR, 0.58; 95% CI, 0.42-0.80; *P* for trend = .001). This association was not changed after adjusting for clinical variables and many other health behaviors (model 2 in Table 2). However, after adjustment for total carbohydrate intake, there was no association in our final multivariable model (model 3). Participants in the fourth quartile had a lower incidence rate for cancer recurrence or death compared with participants in the first quartile, but the difference was not significant (HR, 0.84; 95% CI, 0.58-1.23; *P* for trend = .33).

**Table 2.** Relative Risk of Cancer Recurrence or Death From Any Cause (Disease-Free Survival) and Overall Mortality Among 1011 Patients With Stage III Colon Cancer, by Postdiagnostic Intake of Unprocessed Red Meat and Processed Meats

Type of meat and event	Quartile of intake				*P* for trend^a^
	1	2	3	4	
Unprocessed red meat, median (IQR), No. of servings/wk	1.5 (1.1-1.8)	2.6 (2.3-2.9)	4.0 (3.5-4.6)	6.9 (6.0-8.3)	NA
Recurrence or death					
Events, No.	114	101	93	78	NA
Model, HR (95% CI)					
1^b^	1 [Reference]	0.83 (0.63-1.08)	0.72 (0.54-0.96)	0.58 (0.42-0.80)	.001
2^c^	1 [Reference]	0.82 (0.62-1.09)	0.70 (0.52-0.94)	0.53 (0.38-0.75)	<.001
3^d^	1 [Reference]	0.97 (0.73-1.29)	0.92 (0.67-1.26)	0.84 (0.58-1.23)	.33
Overall mortality					
Events, No.	96	77	72	60	NA
Model, HR (95% CI)					
1^b^	1 [Reference]	0.75 (0.55-1.01)	0.66 (0.48-0.90)	0.54 (0.38-0.77)	.001
2^c^	1 [Reference]	0.76 (0.56-1.05)	0.63 (0.46-0.88)	0.48 (0.33-0.70)	<.001
3^d^	1 [Reference]	0.88 (0.63-1.21)	0.80 (0.57-1.13)	0.71 (0.47-1.07)	.11
Processed meat, median (IQR), No. of servings/wk	0.7 (0.4-0.9)	1.5 (1.3-1.7)	2.7 (2.3-3.2)	5.2 (4.5-6.9)	NA
Recurrence or death					
Events, No.	95	104	94	93	NA
Model, HR (95% CI)					
1^b^	1 [Reference]	1.09 (0.82-1.44)	0.90 (0.67-1.20)	0.95 (0.70-1.29)	.51
2^c^	1 [Reference]	1.03 (0.77-1.37)	0.81 (0.60-1.10)	0.82 (0.59-1.14)	.15
3^d^	1 [Reference]	1.14 (0.85-1.53)	0.96 (0.70-1.30)	1.05 (0.75-1.47)	.94
Overall mortality					
Events, No.	78	79	72	76	NA
Model, HR (95% CI)					
1^b^	1 [Reference]	1.02 (0.74-1.39)	0.85 (0.61-1.18)	0.93 (0.66-1.31)	.58
2^c^	1 [Reference]	0.97 (0.70-1.34)	0.78 (0.56-1.10)	0.82 (0.57-1.17)	.24
3^d^	1 [Reference]	1.07 (0.77-1.48)	0.90 (0.63-1.27)	1.04 (0.72-1.51)	.95

Abbreviations: HR, hazard ratio; NA, not applicable.

^a^ *P *for trend was calculated by modeling the median of each category as a continuous term.

^b^ Cox proportional hazards regression model was adjusted for age (years), sex (male vs female), race (Black, White, or other [ie, Hispanic, Asian, Native Hawaiian, Native American, Indian, other, or multiple race]), and energy intake (kilocalories per day). Three individuals with unknown race were categorized as other race.

^c^ Cox proportional hazards regression model was adjusted for variables in model 1 plus T stage (T1-T2, T3-T4, or missing), number of positive lymph nodes (1-3, ≥4, or missing), baseline performance status (0, 1-2, missing), treatment group (fluorouracil plus leucovorin vs irinotecan, fluorouracil, and leucovorin), body mass index (weight in kilograms divided by height in meters squared), physical activity (metabolic equivalent task hours per week), smoking (never, current, past, or missing), aspirin use (yes vs no), alcohol (grams per day), dark meat fish (yes vs no), vitamin D (International Units per day), cereal fiber (grams per day), high-fat dairy (servings per day), and eggs (servings per day).

^d^ Cox proportional hazards regression model was adjusted for variables in model 2 plus carbohydrates (grams per day).

Results were similar when examining unprocessed red meat in association with overall mortality (Table 2). In the first model adjusted for age, sex, race, and energy intake, participants in the fourth quartile of unprocessed red meat intake had a 46% lower risk of overall mortality compared with participants in the lowest quartile (HR, 0.54; 95% CI, 0.37-0.77; *P* for trend = .001). After adjusting for clinical variables and many other health behaviors, the HR was not meaningfully changed. However, the addition of total carbohydrates (grams per day) to the model attenuated the results and there was no association. In the final multivariable model (model 3), the HR for overall mortality comparing participants in the fourth quartile with participants in the first quartile was 0.71, but the difference was not significant (95% CI, 0.47-1.07; *P* for trend = .11).

We observed no significant associations between processed meat intake and colon cancer recurrence or survival (Table 2). The HR comparing the risk of cancer recurrence or death in the fourth vs first quartile of processed meat was 0.95 (95% CI, 0.70-1.29) in model 1 and 1.05 (95% CI, 0.75-1.47) in the fully adjusted model 3. These values were similar for overall mortality (model 1, HR, 0.93; 95% CI, 0.66-1.31; model 3, HR, 1.04; 95% CI, 0.72-1.51).

There was no evidence of modification by age, sex, or BMI (all *P *for interaction > .05). Furthermore, results did not change when excluding people who experienced recurrent disease or died up to 6 months after the FFQ (data not shown). When we started follow-up at the second FFQ, postdiagnostic intake of unprocessed red meat was significantly inversely associated with risk of cancer recurrence or death, even with adjustment for total carbohydrates (adjusted HR, quartile 4 vs quartile 1, 0.57; 95% CI, 0.34-0.95). Processed meat intake remained unassociated with risk of cancer recurrence or death in this sensitivity analysis.

## Discussion

In this prospective cohort study of 1011 patients with stage III colon cancer, intake of either unprocessed red meat or processed meat after diagnosis was not significantly associated with risk of colon cancer recurrence or mortality. This finding is an important contribution to the literature on postdiagnostic health behaviors and outcomes among cancer survivors. Although our study has limitations, these findings can potentially inform patient counseling and the development of dietary guidelines specific for cancer survivors.

Our results are consistent with 2 prior studies examining postdiagnostic meat intake, as well as pooled results examining prediagnostic meat intake in association with colorectal cancer mortality.^3,4,13^ McCullough et al^13^ examined red and processed meat intake (combined) in association with colorectal cancer-specific and overall mortality in the Cancer Prevention Study II cohort. In that study,^13^ postdiagnostic intake was not associated with either outcome. However, individuals who reported intake at or above the median both before and after diagnosis had an increased risk of colorectal cancer-specific and overall mortality compared with those with low intake at both time points. Notably, individuals with low intake before diagnosis and high intake after diagnosis had the same risk of mortality as those with low intake at both time points. In a secondary analysis using data from the Nurses’ Health Study, Fung et al^14^ also reported no association between red and processed meat intake (combined) with colorectal cancer–specific or overall mortality. Our study expands on these findings by examining unprocessed red meat and processed meat separately, and the conclusions remain the same: neither unprocessed red meat nor processed meat intake after diagnosis is associated with risk of colon cancer recurrence or mortality.

The ACS recommends limiting red and processed meat, and the guideline score does not differentiate between these 2 meat types.^15^ In contrast, the AICR/WCRF has different recommended intake levels for unprocessed vs processed meat.^2^ Specifically, the AICR/WCRF guidelines recommend limiting red meat consumption to “no more than about 3 portions per week” and to “consume very little, if any, processed meat.”^16^ These differing cut points are based on evidence suggesting that processed meat may be more associated with adverse health outcomes, including colorectal cancer risk, than unprocessed red meat.^17,18^ As described already, our findings do not support that either meat type is associated with risk of recurrence or mortality among patients with stage III colon cancer. Although the HR estimates differed between the meat types (Table 2), given the wide and overlapping 95% CIs, we cannot draw conclusions about differences in the association between unprocessed vs processed meat in association with risk of colon cancer recurrence and mortality from our study.

We were concerned about confounding bias due to carbohydrates because our team has previously reported that total carbohydrate intake was associated with an increased risk of colon cancer recurrence and mortality in this cohort.^19^ In addition, in the present study, both unprocessed red meat and processed meat intakes were moderately correlated with carbohydrates (*r* = 0.41 for unprocessed red meat and *r* = −0.29 for processed meat). Therefore, we adjusted for carbohydrates, as well as several other dietary factors that have been previously reported to be associated with colon cancer recurrence and/or mortality. Interestingly, the HRs were not substantially changed until we adjusted for carbohydrates. After adjustment for carbohydrates, there were no associations. On the basis of the growing, but still limited, evidence from studies of postdiagnostic diet and colon cancer survival, dietary recommendations for colon cancer survivors should emphasize consuming a low glycemic diet rich in vegetables and whole grains that may or may not include meat depending on patient preference.^19,20,21^

### Limitations

This study has limitations that should be considered. This was an observational study examining self-reported dietary behavior; thus, we cannot rule out the possibility of unmeasured or residual confounding, and there is known measurement error in self-reported diet.^22^ In addition, our study only included patients with stage III colon cancer; therefore, our results may not apply to those with stage I to II or IV disease. However, this homogeneity also limits potential confounding by a strong prognostic factor (stage). We did not have a measure of prediagnostic meat intake or information about meat cooking practices. Furthermore, our study population was not racially and ethnically diverse and only included individuals participating in a randomized clinical trial. Additional studies in more racially and ethnically diverse populations are needed.

## Conclusions

In this prospective cohort study among patients with stage III colon cancer, postdiagnostic intake of unprocessed red meat or processed meat was not associated with risk of colon cancer recurrence or mortality.
